# Is it aortic stenosis or left ventricular outflow tract obstruction? The complementary role of four-dimensional flow cardiac magnetic resonance imaging

**DOI:** 10.1093/ehjcr/ytad436

**Published:** 2023-09-01

**Authors:** Hosamadin Assadi, Rimma Hall, Pankaj Garg

**Affiliations:** NHS Foundation Trust, Norfolk and Norwich University Hospitals, Colney Lane, Norwich, Norfolk NR4 7UY, UK; Norwich Medical School, University of East Anglia, Norwich Research Park, Norfolk NR4 7UQ, UK; NHS Foundation Trust, Norfolk and Norwich University Hospitals, Colney Lane, Norwich, Norfolk NR4 7UY, UK; NHS Foundation Trust, Norfolk and Norwich University Hospitals, Colney Lane, Norwich, Norfolk NR4 7UY, UK; Norwich Medical School, University of East Anglia, Norwich Research Park, Norfolk NR4 7UQ, UK

Trans-thoracic echocardiography is the primary imaging modality for diagnosing any left ventricular outflow tract obstruction (LVOTO), estimating haemodynamic severity and timing of surgical intervention.^1^ Multi-parametric cardiac magnetic resonance (CMR) imaging provides additional details on tissue characterization and cardiac haemodynamics. We present a case of a 74-year-old gentleman with a history of hypertrophic cardiomyopathy (HCM), where four-dimensional flow (4D flow) identified the mechanism of obstruction in the outflow tract when Doppler methods proved challenging.

## Case description

A 74-year-old gentleman with a history of HCM presented with non-specific abdominal pain. His angiogram and Fabry’s screening were negative. Echocardiography demonstrated left ventricular (LV) hypertrophy with preserved ejection fraction. There was early diastolic dysfunction. Systolic anterior mitral valve leaflet (AMVL) motion causing possible LV outflow tract (LVOT) obstruction was observed on colour-Doppler. Due to the aliasing of the mitral regurgitation (MR) jet, measurement of the direct LVOT gradient proved challenging.

**Figure 1:**
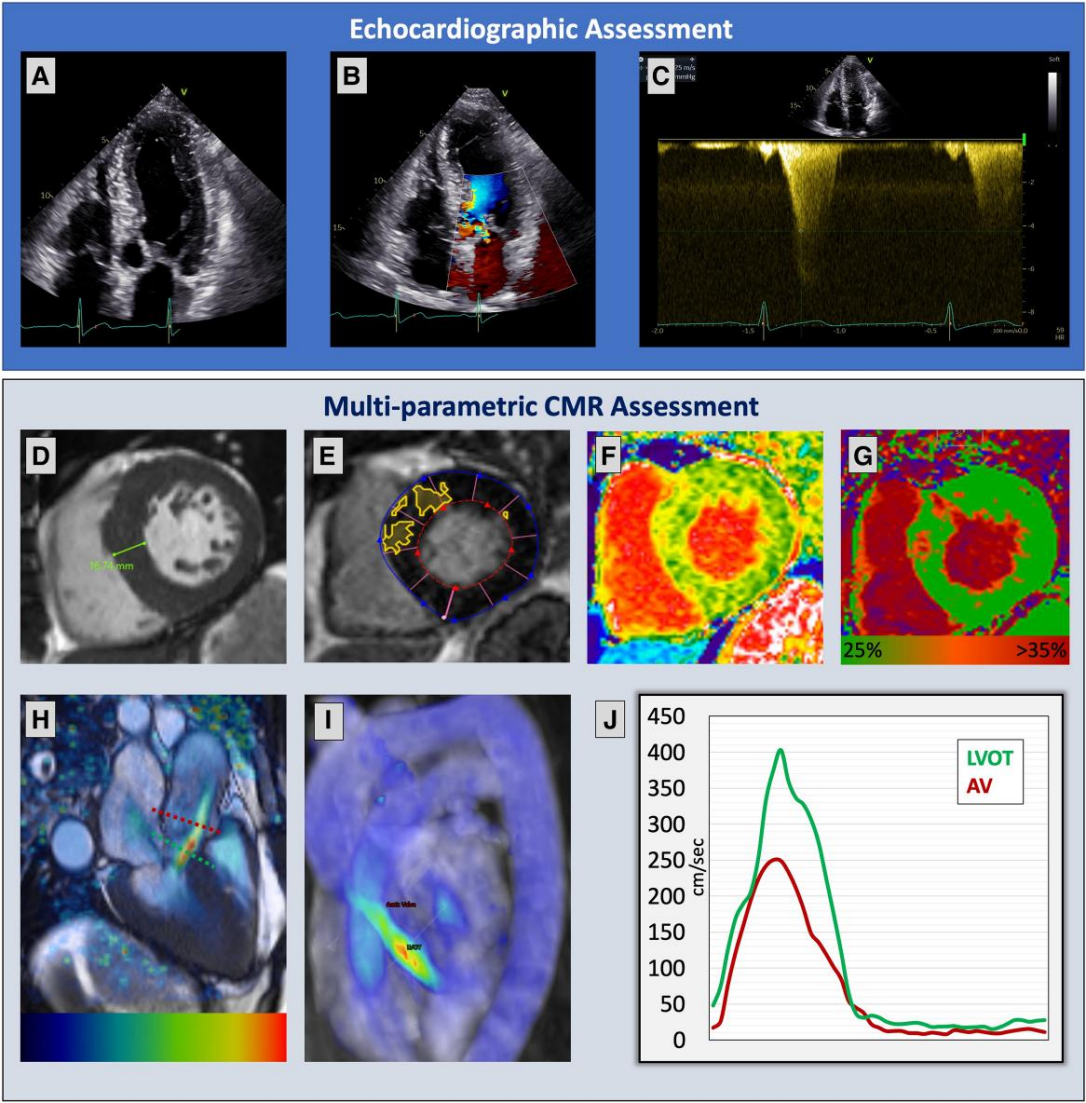
Echocardiographic and multi-parametric cardiovascular magnetic resonance imaging assessment of aortic stenosis. (*A*–*C*) Apical four-chamber long-axis view demonstrating the systolic anterior motion of the anterior mitral valve leaflet towards the outflow tract and possible flow acceleration on colour-Doppler. The peak velocity on continuous wave Doppler was 4.25 m/s. However, due to the mitral regurgitation jet superimposing the outflow tract flow, it was not clear if this was indeed in the outflow tract. Importantly, as this was not a pulse wave Doppler, it was difficult to determine the spatial location of the peak velocity. (*D*) Steady-state free precession short-axis cine images showing asymmetrical septal hypertrophy (16.74 mm at end-diastole). (*E*) Late gadolinium enhancement imaging with a predefined signal threshold of 3 SD, demonstrating mid-wall myocardial fibrosis. (*F* and *G*) Native and post-contrast T1 mapping demonstrating fibrotic changes in the anterior wall and septum, consistent with the diagnosis of hypertrophic cardiomyopathy. (*H*–*J*) Four-dimensional flow three-dimensional velocity visualization of flow and its speed demonstrate that the flow acceleration is happening in the left ventricular outflow tract with a peak velocity of 4.07 m/s. The systolic anterior motion can be seen in (*H*) clearly with resultant mitral regurgitation.

A CMR assessment with 4D flow was performed. There was asymmetrical left ventricular hypertrophy (septum: 16.7 mm) with mid-wall myocardial fibrosis on late gadolinium enhancement, confirmed on T1 mapping. On cines and 4D flow, three-dimensional velocity-mapping, systolic anterior motion (SAM) of the AMVL was seen. The AMVL drift resulted in mild MR, which was directly quantified by the 4D flow. Furthermore, SAM caused LVOTO with a peak resting gradient of 64 mmHg (*Figure 1*; Supplementary material online, *Videos S1–S3*).

Two-dimensional phase-contrast (2DPC) assessment is routinely used in CMR for flow assessment. However, it can miss recognizing the true peak velocity plane, and it can significantly underestimate the true peak velocity, as it captures only the through-plane velocities and not velocities in all directions.^2^ Previous studies have shown how 4D flow can be used for LVOTO pressure gradient assessment.^3^

This specific case not only emphasizes the significance of quantifying peak velocity in the LVOTO using 4D flow but also showcases its visualization capabilities (*Video S4*), which offer precise identification of flow acceleration where routine Doppler or 2DPC techniques are challenging. Four-dimensional flow three-dimensional velocity assessment allowed us to distinctly demonstrate the mechanism of obstruction in the outflow tract and accurately quantify LVOT gradient in a patient with HCM. Hence, this case highlights that 4D flow can complement routine multi-parametric CMR.

## Supplementary Material

ytad436_Supplementary_Data

## Data Availability

No new data were generated or analysed in support of this research.

## Supplementary material

**Consent:** The authors confirm that written consent for submission and publication of this case report, including images and associated text, has been obtained from the patient in line with COPE guidance.

**Funding:** P.G. is funded by the Wellcome Trust (220703/Z/20/Z). For the purpose of Open Access, the author has applied a CC BY public copyright licence to any Author Accepted Manuscript version arising from this submission. The funder had no role in study design, data collection and analysis, publication decisions, or manuscript preparation.
